# Analysis of the fluid contents of simple bone cyst in the mandible

**DOI:** 10.1038/s41598-022-13264-4

**Published:** 2022-06-16

**Authors:** So-Young Choi, Obida Boboeva, Ji Yeon Ham, Chang-Hyeon An, Sung-Tak Lee, Jin-Wook Kim, Seo-Young An

**Affiliations:** 1 Department of Oral and Maxillofacial Surgery, School of Dentistry, Kyungpook National University, Daegu, South Korea; 2 Department of Clinical Pathology, School of Medicine, Kyungpook National University, Kyungpook National University Chilgok Hospital, Daegu, South Korea; 3 Department of Oral and Maxillofacial Radiology, School of Dentistry, IHBR, Kyungpook National University, 2177 Dalgubeol-daero, Jung-gu, Daegu, 41940 South Korea

**Keywords:** Biochemistry, Medical research

## Abstract

Description of simple bone cyst (SBC) content has been controversial. This study aimed to assess and give a clearer picture of the SBC cavity contents. Between 2014 and 2016, 19 patients with SBC verified by histopathological examination were included in this study. SBC cavity content was investigated using clinical, radiographic, surgical, and laboratory findings. The difference in components among cavity fluid, blood, and serum was evaluated using a paired sample t-test for statistical analysis. All 19 SBC cases radiographically and surgically revealed a fluid-filled cavity. The patients’ average age was 21.3 ± 13.2 years, with no sex predominance found. SBCs were found mostly in the anterior mandible (n = 12, 63.2%). All lesions were filled with clear straw-colored or blood-colored floods with low concentration. Although the fluid components were similar to those in the blood and serum in the laboratory analysis, the statistical analysis revealed that the fluid components were not significantly different only for eosinophil (p = 0.43) and basophil (p = 0.06) counts as blood components and sodium (p = 0.76), potassium (p = 0.08), and chloride (p = 0.13) concentration as serum components. The results show that SBC is a fluid-filled cavity, with the cavity fluid being more likely similar to serum rather than blood regarding internal components.

## Introduction

As defined, a simple bone cyst (SBC) is a benign bone lesion that lacks epithelial lining, so called *pseudocyst*, with the cavity being either empty or filled with fluid^1–3^. Traumatic bone cyst, solitary bone cyst, hemorrhagic bone cyst, extravasation cyst, unicameral bone cyst, and idiopathic bone cavity have all been used in the literature to describe the lesion^2,4,5^. According to studies, SBC is more common in the second decade of life^6,7^. The sex and site distribution of SBC is quite controversial, in which female predominance and involvement in the posterior region of the mandible have been documented^3,6,8^. However, Chranovich and Gomez^6^ observed in a recent study that SBC is equally distributed between the two sexes. SBC has been reported in the anterior region of the mandible by several authors^1,7^. In the majority of cases, this cyst is discovered accidentally due to the absence of any clinical symptoms. SBC in the jaw is usually detected by panoramic radiography during routine dental examinations. SBCs are characterized as areas of well-defined unilocular radiolucency with typical interdental scalloping on radiographs^5^.

Furthermore, a lot of conflicting information about the content of the SBC cavity exist. Some authors believed that cavities are always filled with fluid, whereas others argued that an empty cyst cavity containing air might exist^9^. The fluid’s nature was also described as serous or bloody^4,8,10^. The question of whether SBC cavities contain blood or serum may arise at this time. Therefore, we collected and analyzed data of 19 patients diagnosed with SBC in our clinic between 2014 and 2016. We sought to examine the SBC cavity content compared with the components of blood and serum of the same patient and to give more comprehensive insights into SBC of the jawbone.

## Materials and methods

### Subjects

From June 2014 to May 2016, 21 of the 27 patients diagnosed with SBCs volunteered to participate in this study. However, two patients were excluded due to a lack of fluids necessary for analysis. Hence, 19 patients were included in this study. This study was reviewed and approved by the institutional review board of Kyungpook National University Hospital (KNUH 2014-01-023) and was in compliance with the Helsinki Declaration.

**Figure 1:**
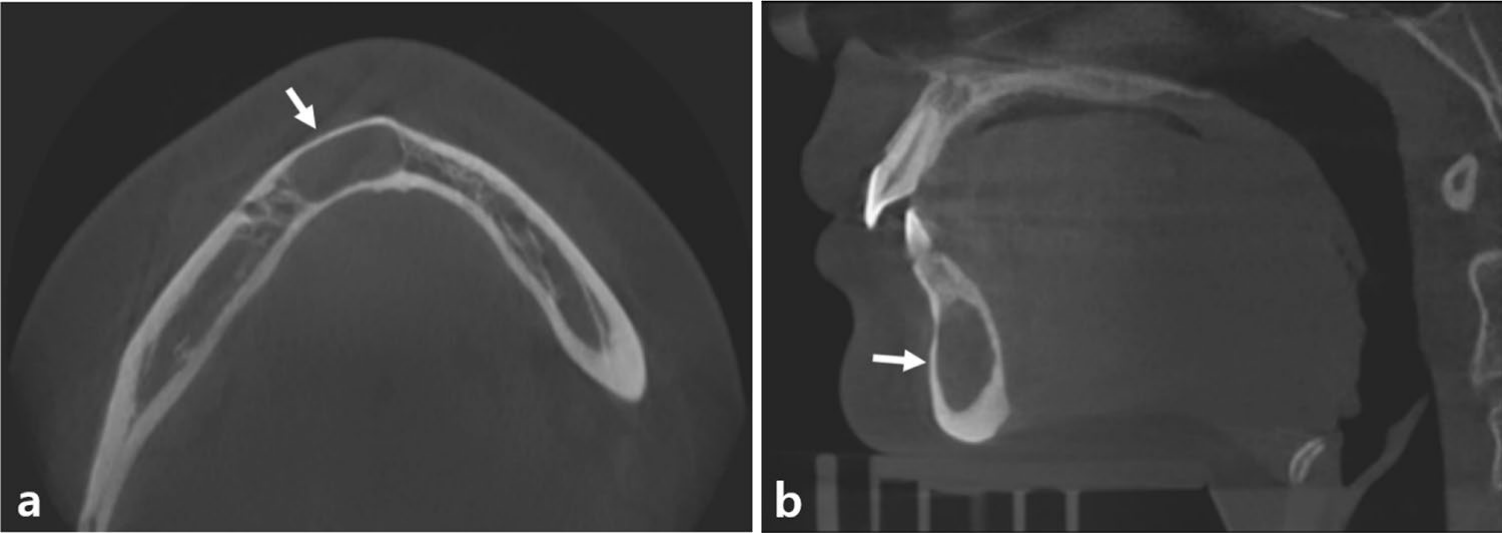
Cone-beam computed tomographic images. Axial (**a**) and sagittal (**b**) images show simple bone cyst in the anterior mandible (white arrow). The cavity is entirely filled with the fluid without aeration, and thinning of the buccolingual cortex is observed.

Before evaluation, all participants were informed of the study’s goals and procedures, as well as signed written consent. Relevant data were retrieved from the electronic database. Age, sex, history of trauma, presence of any symptoms, and cyst location were among the clinical data. For the radiographic examination, the results of cone-beam computed tomography (CBCT) were used (Fig. 1). A radiologist with more than 15 years of expertise evaluated CBCT images on a 21.2-inch WIDE CX30p 3MP Color LED Diagnostic Monitor (WIDE Medical, Gyeonggi-do, Korea). The PACS viewer’s scale tool was used to measure the length, width, and height of axial, coronal, and sagittal images. The images were evaluated twice, at 4-week interval, and the mean value was used (Table 1). The SBC content, complete blood count, and chemical components of the serum were analyzed for the surgical and laboratory data.

**Table 1:** Clinical and radiographic findings of 19 cases with SBC.

Number	Age	Sex	History of trauma	Symptom	Location	Size of cyst (mm)		
						Length	Width	Height
1	22	M	Yes	No	Mn. anterior	15.98	5.14	10.56
2	16	F	Yes	No	Mn. anterior	11.83	9.41	12.4
3	16	F	No	No	Mn. anterior	18.82	8.7	17.44
4	12	M	No	No	Mn. anterior	17.01	8.28	14.95
5	15	F	No	No	Mn. anterior	19.61	5.63	11.92
6	16	F	No	No	Mn. anterior	18.6	7.92	14.83
7	18	M	No	No	Mn. posterior	27.5	12.06	18.74
8	15	M	No	No	Mn. anterior	18.43	10.27	18.53
9	13	M	No	No	Mn. posterior	16.59	9.39	11.4
10	57	F	No	No	Mn. posterior	23.87	19.49	14.92
11	15	F	No	No	Mn. anterior	20.67	9.51	12.32
12	13	M	No	No	Mn. anterior	12.14	6.3	12.15
13	17	M	No	No	Mn. anterior	14.45	9.73	12.42
14	20	M	No	No	Mn. posterior	30.7	11.2	20.03
15	49	F	No	No	Mn. posterior	15.91	16.47	17.32
16	15	F	No	No	Mn. anterior	18.44	9.44	18.89
17	16	M	No	No	Mn. posterior	29.89	12.86	21.37
18	47	F	No	Yes (pain)	Mn. posterior	19.29	14.79	23.23
19	13	F	No	No	Mn. anterior	17.04	6.22	12.63

*SBC* simple bone cyst, *Mn.* Mandible.

### Surgical procedure and laboratory analysis

**Figure 2:**
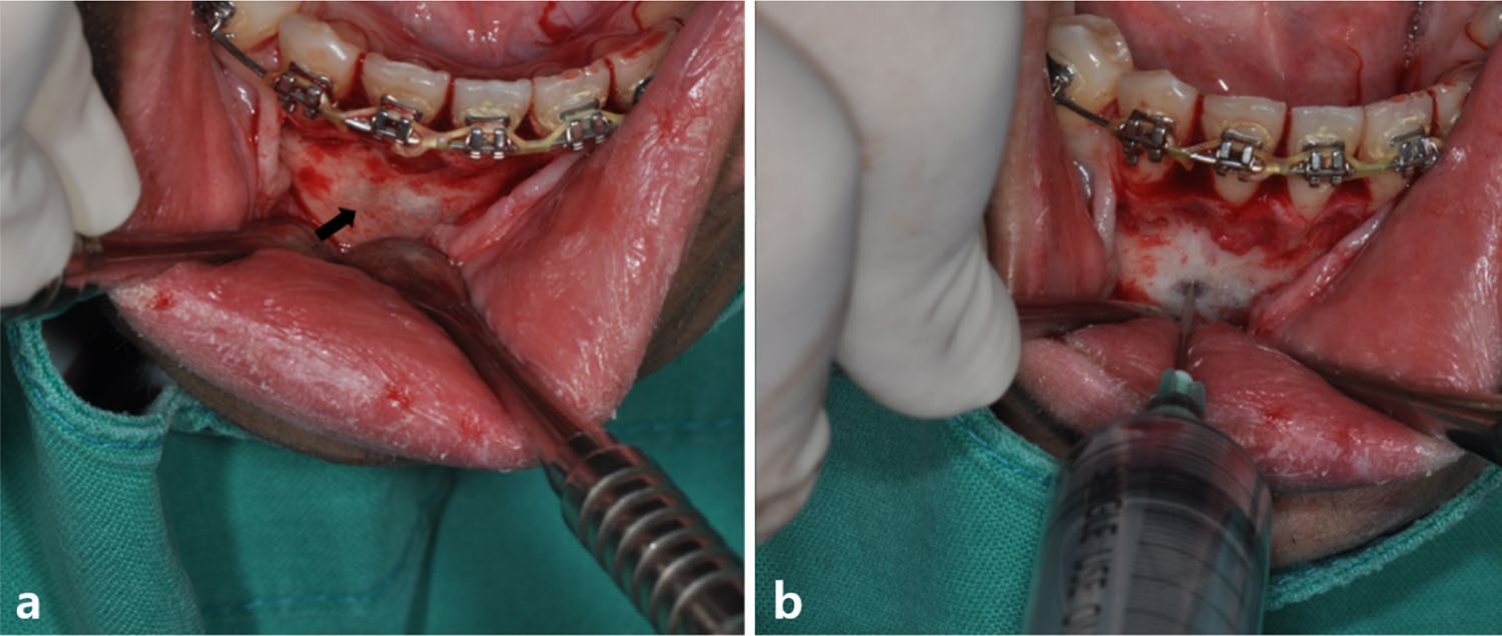
Clinical photograph of surgical procedure. (**a**) Bone exposure. A thin and transparent buccal cortex (black arrow) over the lesion is exposed. (**b**) Fluid collection. A 10 mL syringe is inserted directly to the cavity through the thinnest cortex, and the fluid is collected without any contamination from the extra-lesional blood or saliva.

Surgery was performed by a skilled surgeon with more than 10 years of professional expertise to accurately collect the SBC cavity content. To prevent contamination from surrounding blood or saliva, thorough hemostasis and suction were performed after mucosal incision. Under CBCT guidance, the needle of a 10 mL syringe was introduced through the thinnest cortical bone site, and fluid was aspirated (Fig. 2). If the cortical bone was too thick, a bur was used to carefully grind it down so the needle could be inserted and fluid taken. After collecting the liquid, a 5 mm hole was used to confirm the absence of cystic lining and a sample of the bone and surrounding tissues was taken for biopsy.

For complete blood count (CBC) and white blood cell differential count analyses, preoperative blood samples and collected cyst liquid samples were anticoagulated with dipotassium ethylenediaminetetraacetic acid (EDTA) and evaluated on a Siemens ADVIA® 2120i hematology analyzer (Siemens Healthcare Diagnostics, Erlangen, Germany). Preoperative serum samples were collected using a serum-separating tube (SST) and properly centrifuged, and liquid samples obtained in SST were measured on Cobas® 8000 Modular chemistry analyzer (Roche Diagnostics, Mannheim, Germany) to determine total protein, total cholesterol, sodium, potassium, and chloride levels. Every CBC and chemistry test was determined within 4 h of blood and liquid collection.

### Statistical analysis

Standard software (SPSS, version 25, IBM Corp., Armonk, NY) was used to conduct the statistical analysis. Mean with SD values and range were used for descriptive statistics. The paired sample t-test was used to assess statistical differences between SBC fluid content and blood and serum components. The 5% cut point was set for statistical significance.

### Informed consent statement

Informed consent was obtained for all patients involved in the study including for minors from their parents and/or legal guardians.

## Results

### Subjects

This study evaluated 19 cases. Table 1 shows the clinical and radiographic findings. The mean age of the 19 patients was 21.3 ± 13.2 years (range 12–57 years), with 14 (73.7%) of them in their 20 s. No sex predominance (male-to-female ratio = 9:10) was noted. After routine radiographic examination, 18 (94.7%) asymptomatic cases were detected, while only 1 patient reported pain. Two (10.5%) patients had a history of trauma in the jaw.

All lesions were located in the mandible, with seven (36.8%) cases being found in the mandibular posterior region, while the rest were in the anterior region (n = 12, 63.2%).

### Computed tomography and laboratory analysis

On CBCT imaging, the SBC had a mean anteroposterior length of 19.3 ± 5.1 mm, a mean buccolingual width of 10.1 ± 3.6 mm, and a mean height of 15.6 ± 3.7 mm. All SBCs were unilocular on radiography. SBC manifested as radiolucency with radiopaque mass in all three middle-aged patients. And there was no tooth displacement or resorption.

Radiographically and by needle aspiration, all lesions were filled with fluid. The aspirated fluid was either a straw-colored clear liquid or reddish liquid similar to blood, but with a low concentration.

The internal fluid from the lesion had components similar to those found in the blood and serum, according to the laboratory analysis.

### Statistical analysis of components

Components such as red and white blood cells, hemoglobin, hematocrit, segmented neutrophils, lymphocytes, and monocytes showed notable differences between blood and liquid cavity in the statistical analysis. Lymphocyte and monocyte counts were higher in the cavity liquid, while red and white blood cell counts including hemoglobin, hematocrit, and segmented neutrophils were higher in blood than in the cavity liquid.

Chemical analysis revealed that the number of serum components such as total protein and cholesterol differed significantly from those in the SBC fluid. Total protein was higher in the serum than in the fluid, while total cholesterol was higher in the cavity fluid.

However, only eosinophils (p = 0.43) and basophils (p = 0.06) as blood components, as well as sodium (p = 0.76), potassium (p = 0.08), and chloride (p = 0.13) concentration as serum components, were shown to be substantially different in the cavity fluids. The internal components of SBC cavity fluid, blood, and serum are compared in Tables 2 and 3.

**Table 2:** Component comparison of the blood and SBC fluid contents.

Components (unit)	Blood (mean ± SD)	SBC fluid content (mean ± SD)	p value
White blood cells (× 10^3^/uL)	6.85 ± 1.46	3.26 ± 2.49	0.00
Red blood cells (× 10^6^/uL)	4.77 ± 0.48	2.06 ± 1.51	0.00
Hemoglobin (g/dL)	14.40 ± 1.35	5.93 ± 4.52	0.00
Hematocrit (%)	40.28 ± 6.72	14.69 ± 12.37	0.00
Segmented neutrophils (%)	53.94 ± 10.11	40.57 ± 9.69	0.00
Lymphocytes (%)	34.64 ± 8.79	44.12 ± 11.13	0.00
Monocytes (%)	5.75 ± 1.43	7.47 ± 2.96	0.01
Eosinophils (%)	2.80 ± 1.85	3.46 ± 3.60	0.43
Basophils (%)	0.59 ± 0.22	1.62 ± 2.20	0.06

*SBC* simple bone cyst, *SD* standard deviation.

**Table 3:** Component comparison of the serum and SBC fluid contents.

Components (unit)	Serum (mean ± SD)	SBC fluid content (mean ± SD)	p value
Total protein (g/dL)	7.25 ± 0.52	6.40 ± 0.65	0.00
Total cholesterol (mg/dL)	157.31 ± 35.70	198.28 ± 45.56	0.00
Sodium (mmol/L)	140.95 ± 3.20	140.68 ± 4.82	0.76
Potassium (mmol/L)	7.39 ± 9.11	13.32 ± 19.63	0.08
Chloride (mmol/L)	101.68 ± 3.27	102.89 ± 5.47	0.13

*SBC* simple bone cyst, *SD* standard deviation.

## Discussion

SBC of the jaw is considered rare^4^; therefore, most studies are case reports. Only a few studies have looked at the relationship between SBC of the jaws and its internal fluid components. This study aimed to report clinical, radiographic, surgical, and laboratory findings of 19 patients with SBC, with a particular focus on the components of the cyst liquid contents.

SBC cavity in the jaw is often reported to be empty^1,11–16^. In a recent systematic analysis of SBC of the jaw, more than half of all reported cases have empty cavities^6^. However, the cavity being vacant might be mistaken evaluation^9,10^. All 19 SBC cavities in this study were consistently filled with fluid, as confirmed both radiographically and surgically.

Moreover, researchers suggest that blood or serous fluid is present in the cyst cavity^4,11–14,17,18^. To our knowledge, no studies evaluating internal components of cyst content have found any similarities SBC cavity content and blood. Contamination of the fluid during the creation of a window in the cyst wall is thought to be a possible explanation for the blood-contained SBC cavity fluid. The internal fluid was clear at the onset of needle aspiration, according to our findings. To avoid misdiagnosis owing to a surgical error, thorough suction and chilling were planned. Although some fluid samples included blood, it is believed that blood was drawn off the cavity wall at the end of the aspiration. This study reveals that fluid contents have substantially different concentrations of blood components, except for eosinophils and basophils. Eosinophils and basophils were found in the cytological findings of aspirates from idiopathic bone cysts, according to Rivero et al.^2^. However, there is no clear explanation for this phenomenon. In 47 cases of odontogenic cysts, Madhusudan et al.^19^ explored the role of eosinophils in bone resorption. They believe that a factor secreted by mast cells attracts eosinophils. The exact presence and function of these inflammatory cells in SBCs are still unknown. Despite this, the authors claim that SBC is a fluid-filled cavity that shows no signs of inflammation^20,21^. Although fluid has contained inflammatory cell, this complied with the cases in our research.

The mechanism of cyst formation has been the subject of numerous studies. In the literature, the trauma and hemorrhagic and vascular blockage concepts are most commonly encountered^11–13,15,17,22,23^. The formation of cyst content has been investigated using fluid aspirates. It is believed that fluid aspirates have similar contents to that in serum with respect to proteins and electrolytes^14,22,24^. Although this was in consistent with our findings, the statistical analysis of total protein concentrations in the fluid and serum revealed significant differences (with lower total protein in the fluid). Consequently, studies have suggested that the SBC fluid content represents transudate (contains lower total protein than serum)^4,18,24,25^. Accordingly, plasma diffusion may have a role in the formation of SBC fluid contents, according to Skaug^24^. Suzuki^26^ investigated the fluid components of jaw cysts, other than bone cysts, and proposed an electrolyte diffusion mechanism from serum through the cyst wall.

The majority of SBCs (14 of 19 cases) were found in patients in their second decade of life, with similar prevalence in both sexes^6^. In this study, 18 cases were detected after routine radiographic examination. This supports prior observations that most SBCs are asymptomatic^6^.

Lesions are commonly found in the mandible^6^, which correlates with our findings. Although the posterior mandible is the most common location^3,4^, the anterior mandible was most frequently implicated (12 of 19 cases) in the current study.

A combination of cemento-osseous dysplasia with SBC was notably present in three middle-aged female patients. This was backed up by prior reports^10,27,28^.

The study’s drawback can be attributed to the small number of participants. Therefore, future research should focus on SBC fluid contents in a larger number of cases.

## Conclusions

During radiographic examination and surgery, all SBC cavities were filled with fluid. The results reveal that SBC cavities are filled with serum-like fluid rather than blood. The nature of SBC fluid contents may have been described incorrectly in the literature, at least in cases where an accurate aspiration procedure was not performed. The use of a meticulous aspiration technique to obtain true and non-contaminated fluid is proposed to explain the nature of the fluid contents.

## Data Availability

The data presented in this study are available on request from the corresponding author. The data are not publicly available due to the founder’s policy.
